# Integrated Bioinformatics Analysis of Master Regulators in Anaplastic Thyroid Carcinoma

**DOI:** 10.1155/2019/9734576

**Published:** 2019-04-28

**Authors:** Zongfu Pan, Lu Li, Qilu Fang, Yangyang Qian, Yiwen Zhang, Junfeng Zhu, Minghua Ge, Ping Huang

**Affiliations:** ^1^Department of Pharmacy, Zhejiang Cancer Hospital, Hangzhou 310022, China; ^2^Key Laboratory of Head & Neck Cancer Translational Research of Zhejiang Province, Zhejiang Cancer Hospital, Hangzhou 310022, China; ^3^Department of Pharmacy, The First Affiliated Hospital, College of Medicine, Zhejiang University, Hangzhou 310003, China

## Abstract

Anaplastic thyroid carcinoma (ATC) is one of the most aggressive and rapidly lethal tumors. However, limited advances have been made to prolong the survival and to reduce the mortality over the last decades. Therefore, identifying the master regulators underlying ATC progression is desperately needed. In our present study, three datasets including GSE33630, GSE29265, and GSE65144 were retrieved from Gene Expression Omnibus with a total of 32 ATC samples and 78 normal thyroid tissues. A total of 1804 consistently changed differentially expressed genes (DEGs) were identified from three datasets. KEGG pathways enrichment suggested that upregulated DEGs were mainly enriched in ECM-receptor interaction, cell cycle, PI3K-Akt signaling pathway, focal adhesion, and p53 signaling pathway. Furthermore, key gene modules in PPI network were identified by Cytoscape plugin MCODE and they were mainly associated with DNA replication, cell cycle process, collagen fibril organization, and regulation of leukocyte migration. Additionally, TOP2A, CDK1, CCNB1, VEGFA, BIRC5, MAPK1, CCNA2, MAD2L1, CDC20, and BUB1 were identified as hub genes of the PPI network. Interestingly, module analysis showed that 8 out of 10 hub genes participated in Module 1 network and more than 70% genes of Module 2 consisted of collagen family members. Notably, transcription factors (TFs) regulatory network analysis indicated that E2F7, FOXM1, and NFYB were master regulators of Module 1, while CREB3L1 was the master regulator of Module 2. Experimental validation showed that CREB3L1, E2F7, and FOXM1 were significantly upregulated in ATC tissue and cell line when compared with normal thyroid group. In conclusion, the TFs regulatory network provided a more detail molecular mechanism underlying ATC occurrence and progression. TFs including E2F7, FOXM1, CREB3L1, and NFYB were likely to be master regulators of ATC progression, suggesting their potential role as molecular therapeutic targets in ATC treatment.

## 1. Introduction

Thyroid cancer is one of the most common cancers with 567,000 new cases worldwide in 2018 [1]. Anaplastic thyroid carcinoma (ATC) is the most aggressive type of thyroid cancer. It is responsible for more than half of all thyroid cancer deaths, despite only accounting for 2% of thyroid cancer incidence. The overall survival rate of this undifferentiated thyroid cancer is as low as 13% [2]. ATC grows rapidly and exhibits highly invasive behaviour, with 40% of ATC patients suffering extrathyroidal extension and lymph node metastasis, whereas the remaining 60% of patients have distant metastases [3]. Although certain novel treatment methods, including surgical resection, radioiodine ablation, chemotherapy, and molecular targeted therapy, provide possibilities for the treatment of ATC, there is limited survival improvement over the last decades. Therefore, identification of regulatory networks associated with ATC and investigation of emerging molecularly targeted therapies have been an ongoing interest.

In the past couple of years, considerable advances have been made in demonstrating the genomic and transcriptomic landscape of ATC. Landa et al. [4] reported that ATC bore a high mutation burden and probably arose from preexisting differentiated thyroid cancer (DTC) through the accumulation of key additional genetic abnormalities. Other molecular alterations like microRNAs (miRNAs) expression are also observed in ATC. In particular, miR-200, miR-30, let-7d, and let-7g are downregulated in ATC, and others, such as miR-146, miR-221, miR-222, and the miR-17-92 cluster, are upregulated [5, 6]. By performing mRNA expression microarray, Hébrant et al. found much stronger epithelial-mesenchymal transition (EMT), dedifferentiation, and glycolytic phenotypes showed by the ATC [7]. Although these studies revealed dysregulation of key signals in ATC, the master regulators underlying ATC progression are still far from clear.

High-throughput gene profiling comparing cancer to control has been a powerful tool in revealing pivotal pathways. Through transcriptomics data mining, the landscape of tumor biology and malignant signatures were gradually uncovered. Transcription factors (TFs) are known to play master role in tumorigenesis and tumor progression by widely promoting or blocking the transcription of their targets, while they are often “buried” in the middle of the data as not rank in the “top” list either by* P* value or by fold change. Identifying TFs of malignant signatures could provide a comprehensive view in interpretation of tumor biology. Though TFs activation is regulated by many factors more important than their expression levels such as protein localization or protein post-translational modifications, the transcriptomics mining of TFs offers a potential direction for future mechanism research.

Individual study often suffered from lack of reproducibility. To overcome this shortage, we tried to find differentially expressed genes (DEGs) and master regulators that consistently changed in ATC tumors via analyzing gene microarrays from three studies (GSE33630, GSE29265, and GSE65144) in the present study. The commonly changed DEGs were selected for gene function annotation and pathway enrichment. Moreover, protein-protein interaction network and TFs regulatory network were constructed to identify key gene modules and master regulators in ATC. By combining integrated bioinformatics analysis with experimental validation, we found several novel TFs that were tightly relevant to aggressiveness of ATC and may be potential targets for ATC therapy.

## 2. Materials and Methods

### 2.1. Microarray Data

Three microarray datasets (GSE33630, GSE29265, and GSE65144) containing anaplastic thyroid carcinomas (ATC) and normal thyroid tissues were retrieved from Gene Expression Omnibus (GEO, http://www.ncbi.nlm.nih.gov/geo/) database in the National Center for Biotechnology Information (NCBI). The microarray dataset of GSE33630 included 11 ATC and 45 normal thyroids [8]. The GSE29265 dataset contained 9 ATC and 20 normal thyroids, and the GSE65144 had 12 ATC and 13 normal thyroids [9]. All the datasets were based on GPL570 (Affymetrix Human Genome U133 Plus 2.0 Array), which contains 54,675 probes.

### 2.2. Data Processing and Screening of Differentially Expressed Genes (DEGs)

The Relative Log Expression (RLE) plots were conducted and samples in each dataset centered near 0 and had similar spread, which indicated that changes between samples were low and these datasets were sufficient for normalization and statistical analysis (Figures S1–S3). The GEO2R web tool (http://www.ncbi.nlm.nih.gov/geo/geo2r) was applied to identify the DEGs between ATC and normal thyroid samples in three datasets, respectively. GEO2R is an online tool that allows users to perform comparisons between different groups in GEO series, which depends on the GEOquery and the Linear Models for Microarray Analysis (LIMMA) R packages [10, 11]. Significant DEGs were identified by empirical Bayes test. To control the False Discovery Rate (FDR), Benjamini and Hochberg method was applied to adjust the* P* values for multiple testing. The thresholds for filtering DEGs were set as FDR<0.05 and |log_2_ fold change (FC)|≥ 1. Consistently changed DEGs from three datasets were identified by the Venny 2.1 online tool (http://bioinfogp.cnb.csic.es/tools/venny).

### 2.3. Function Annotation and Pathway Enrichment of DEGs

Gene ontology (GO) and Kyoto Encyclopedia of Genes and Genomes (KEGG) were applied for the function annotation and pathway enrichment analysis of DEGs through using the Database for Annotation Visualization and Integrated Discovery (DAVID; https://david.ncifcrf.gov) [12]. Adjusted* P* values were calculated by Benjamini and Hochberg method and the thresholds were set as FDR<0.05 to indicate a statistically significant difference.

### 2.4. Protein-Protein Interaction (PPI) Network Construction and Analysis of Modules

The online database Search Tool for the Retrieval of Interacting Genes (STRING, http://string-db.org) is widely used to identify the interactions between known proteins and predicted proteins and to construct a PPI network [13]. The common DEGs among three datasets were inputted into STRING to construct PPI network and visualized by Cytoscape software (version 3.6.1). To identify the hub genes and key modules, the Cytoscape plugin Molecular Complex Detection (MCODE) was applied to conduct module analysis in the resulting PPI network [14]. Degree cut-off was set as 5, and the rest parameters were set as default. Moreover, plugin ClueGO was employed to create pathway interaction network and to annotate the function of key modules [15].

### 2.5. Master Regulator Analysis

In order to characterize regulatory networks underlying ATC, the Cytoscape plugin iRegulon was used to identify master regulators that targeted key modules above. The master regulators were transcription factors whose transcriptional target sets are highly overlapping with the observed gene signatures. The algorithm was based on a typical ranking-and-recovery strategy, which was previously described by Janky et al. [16]. All the default parameters were left unchanged when predicting TFs. Then, TFs that covered more than 50% of gene sets or normalized enrichment score (NES) >5 were selected to construct the regulatory network.

### 2.6. Validation of Master Regulator Expression

Three human ATC tissues and six nontumorous tissues were obtained from patients who underwent surgical resection at Zhejiang Cancer Hospital. All specimens had a pathological diagnosis at the time of assessment. Studies were approved by the Ethics Committee of Zhejiang Cancer Hospital.* In vitro* experiments were conducted in 8505C (DSMZ, Cat# ACC-219), 8305C (DSMZ, Cat# ACC-133), CAL62 (DSMZ, Cat# ACC-448), and Nthy-ori 3-1 (ECACC, Cat# 90011609) cell lines. To detect the protein expression of candidate TFs, western blot and immunohistochemistry were employed as previously described [17, 18]. The primary antibodies contained rabbit anti-E2F7 (Proteintech, Rosemont, USA), rabbit anti-FOXM1 (Proteintech, Rosemont, USA), mouse anti-NFYB (Santa Cruz Biotechnology, Dallas, USA), and rabbit anti-CREB3L1 (Abcam, Cambridge, UK). To detect the mRNA expression of candidate TFs, total mRNA of the human specimens and cell samples was extracted by Trizol (Invitrogen, San Diego, CA). Then, RNA was treated by RT reagent kit (TAKARA, Dalian, China). SYBR Premix Ex Taq Kit (TAKARA, Dalian, China) was used for amplifications. All these reactions were carried out in triplicate. The primer sequences were listed as follows: CREB3L1 forward primer GCACCTGGACCACTTTACGG and reverse primer AGCACAGGGTCATCAAAGAAG. E2F7 forward primer AAAGGGACTATTCCGACCCAT and reverse primer ACTTGGATAGCGAGCTAGAAACT. FOXM1 forward primer GGAGCAGCGACAGGTTAAGG and reverse primer GTTGATGGCGAATTGTATCATGG. NFYB forward primer GGTGCCATCAAGAGAAACGG and reverse primer GTGACTGCTCCACCAATTCC. ACTB forward primer AGGGGCCGGACTCGTCATACT and reverse primer GGCGGCACCACCATGTACCCT. The relative expression levels of mRNA were calculated using the 2^−ΔΔCt^ method [19]. Statistical analysis was performed by GraphPad Prism version 6.0. Differences between ATC and normal groups were calculated by two-tailed Student's t-test. Differences between different cell lines were calculated by One-way ANOVA.* P* value <0.05 was considered statistically significant.

## 3. Results

### 3.1. Identification of DEGs in Anaplastic Thyroid Carcinoma (ATC)

**Figure 1:**
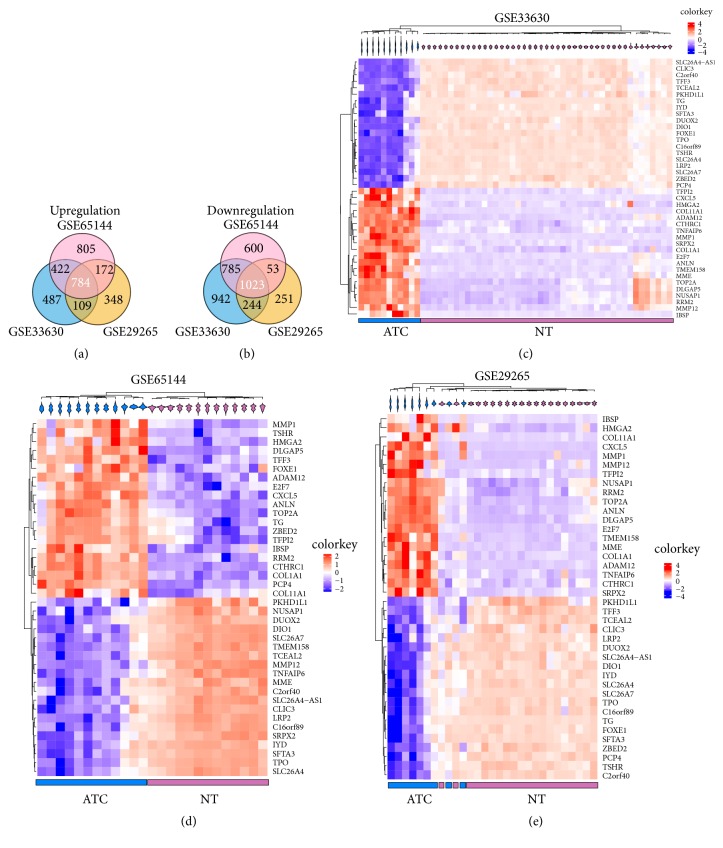
Identification of commonly changed DEGs from the three cohort profile datasets (GSE33630, GSE29265, and GSE65144) and the hierarchical clustering analysis of DEGs. (a-b) Venn diagram displayed consistently up- and downregulated DEGs in three groups. (c–e) Top 20 up- and downregulated DEGs depending on fold changes were hierarchically clustered and displayed by heatmap. Probe values (log2 transformation) of each gene were normalized by row Z-score, respectively. DEGs, differentially expressed genes.

Microarray datasets including GSE33630, GSE29265, and GSE65144 were retrieved from GEO. DEGs (FDR<0.05, |log_2_ FC|≥ 1) of three microarray datasets were screened out basing on the GEO2R analysis, respectively. The percentages of dysregulated genes in GSE33630, GSE29265, and GSE65144 were 20.4%, 12.7%, and 19.7%, respectively. A total of 1807 consistently expressed genes (784 upregulated and 1023 downregulated genes) were identified by the intersection of DEGs from three datasets (Figures 1(a)-1(b)). Then, mean fold changes of consistently changed DEGs from three datasets were calculated. The top 20 up- and downregulated DEGs were hierarchically clustered and displayed as heatmap in Figures 1(c)–1(e), respectively. As the results showed in Figures 1(c)–1(e), these top 40 DEGs could clearly distinguish ATC from normal thyroid tissues.

### 3.2. Function Annotation and Pathway Enrichment of DEGs

To investigate the biological function of DEGs between ATC and normal tissues, GO and KEGG analyses were performed using the DAVID online analysis tool.

**Figure 2:**
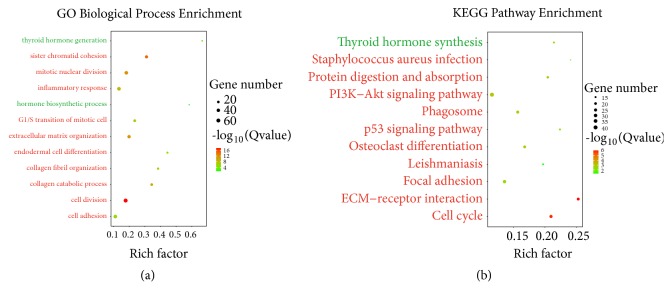
Enriched GO (Biological Process) terms and KEGG pathways of upregulated and downregulated DEGs. For upregulated DEGs, top ten significantly enriched GO terms and KEGG pathways were displayed, respectively. GO, gene ontology; KEGG, Kyoto Encyclopedia of Genes and Genomes; DEGs, differentially expressed genes.

Basing on the GO biological process (BP) enrichment (Figure 2(a)), the upregulated genes were mainly associated with cell division, sister chromatid cohesion, mitotic nuclear division, extracellular matrix organization, collagen catabolic process, inflammatory response, G1/S transition of mitotic cell, collagen fibril organization, cell adhesion, and endodermal cell differentiation. Function annotation of downregulated DEGs by GO BP indicated that thyroid hormone generation and hormone biosynthetic process were significantly enriched (Figure 2(a)).

Subsequently, KEGG pathway analysis demonstrated that the upregulated DEGs were mainly enriched in key pathways including ECM-receptor interaction, cell cycle, PI3K-Akt signaling pathway, protein digestion and absorption, phagosome, osteoclast differentiation, focal adhesion, p53 signaling pathway, staphylococcus aureus infection, and leishmaniasis (Figure 2(b)). The downregulated DEGs significantly enriched in thyroid hormone synthesis (Figure 2(b)).

### 3.3. Protein-Protein Interaction (PPI) Network Construction

**Figure 3:**
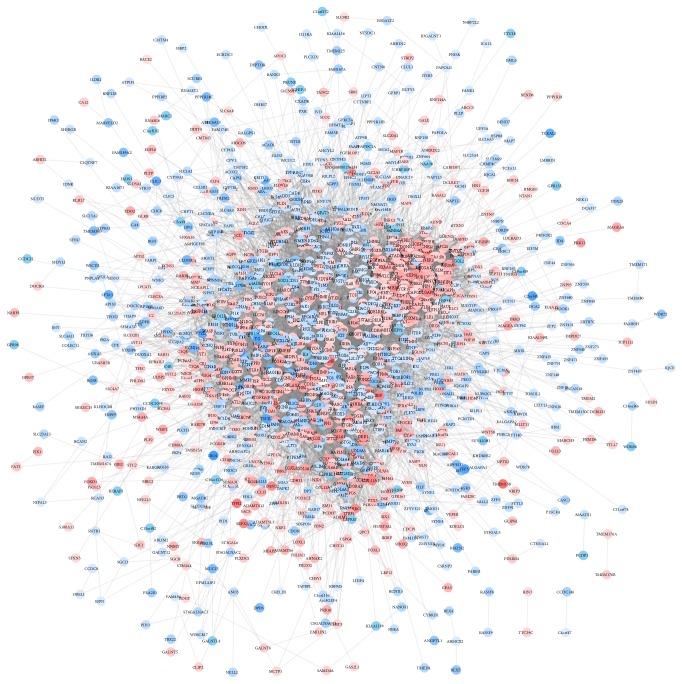
The protein-protein interaction network of DEGs. The PPI network was constructed by STRING and visualized by Cytoscape software. Red nodes represented upregulated DEGs. Blue nodes represented downregulated DEGs. DEGs, differentially expressed genes; PPI, protein-protein interaction; STRING, Search Tool for the Retrieval of Interacting Genes.

The online database STRING was applied to construct the PPI network. As shown in Figure 3, the PPI network consisted of 1438 nodes interacting via 15,220 edges. Expression level of upregulated DEGs (red) and downregulated DEGs (blue) in the PPI network was shown using the mean expression value of each gene from three datasets described above. Basing on the PPI network analysis, the top 10 DEGs with highest node degree were regarded as hub genes of ATC. These hub genes were TOP2A (degree=239), CDK1 (degree=194), CCNB1 (degree=170), VEGFA (degree=169), BIRC5 (degree=154), MAPK1 (degree=154), CCNA2 (degree=152), MAD2L1 (degree=151), CDC20 (degree=148), and BUB1 (degree=146).

### 3.4. Modules Analysis of PPI Network

**Figure 4:**
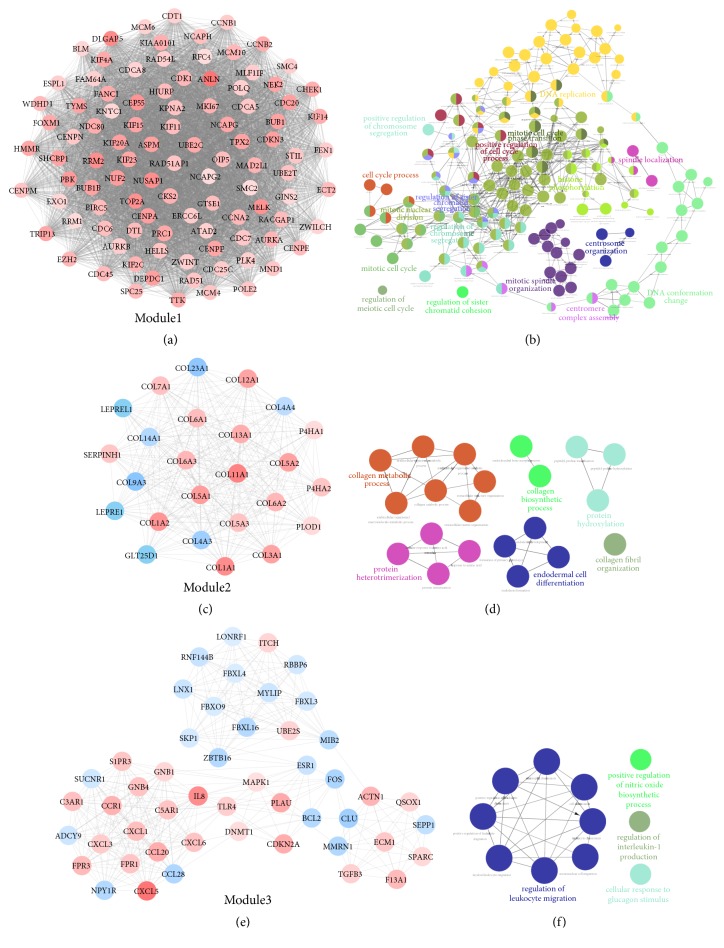
Module analysis of PPI network and gene function annotation. (a, c, e) Three key modules identified from the PPI network. Red nodes represented upregulated DEGs. Blue nodes represented downregulated DEGs. (b, d, f) Pathway interaction network of three gene modules analyzed by ClueGO. PPI, protein-protein interaction.

Further MCODE analysis revealed three key modules from the PPI network. Module 1 consisted of 100 nodes and 4335 edges, which were mainly enriched in DNA replication, spindle localization, mitotic cell cycle phase transition, positive regulation of cell cycle process, positive regulation of chromosome segregation, cell cycle process, histone phosphorylation, regulation of sister chromatid segregation, regulation of chromosome segregation, mitotic nuclear division, mitotic cell cycle, regulation of meiotic cell cycle, regulation of sister chromatid cohesion, mitotic spindle organization, centrosome organization, centromere complex assembly, and DNA conformation change (Figures 4(a)-4(b)). Module 2 consisted of 25 nodes and 285 edges, which were associated with collagen metabolic process, collagen biosynthetic process, collagen fibril organization, protein hydroxylation, protein heterotrimerization, and endodermal cell differentiation (Figures 4(c)-4(d)). Module 3 contained 49 nodes and 330 edges, which mainly participated in regulation of leukocyte migration, positive regulation of nitric oxide biosynthetic process, regulation of interleukin-1 production, and cellular response to glucagon stimulus (Figures 4(e)-4(f)).

### 3.5. Regulatory Network Construction and Master Regulators Identification in ATC

**Figure 5:**
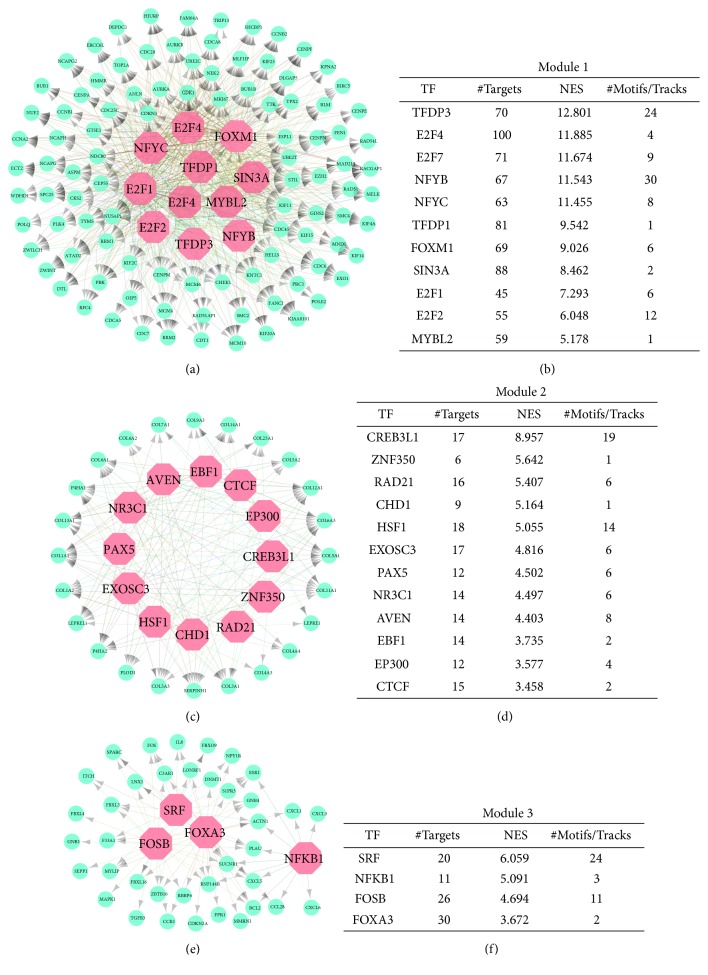
Identification of master regulators in regulatory network of ATC. (a, c, e) Results of the regulatory analysis with iRegulon for three key modules. The cyan nodes represented regulated targets. The light red nodes indicated transcription factors. (b, d, f) Lists of TFs that targeted the genes in three modules. TFs, transcription factors; NES, normalized enrichment score.

To elucidate the potential regulators that targeted the genes of key modules in the PPI network, we queried iRegulon to predict the regulators and targets. In the regulatory network of Module 1, 11 TFs were considered as master regulators (Figures 5(a)-5(b)). TFDP3, E2F4, and E2F7 were the top three enriched regulators with as much as 70, 100, and 71 targets, respectively. In the regulatory network of Module 2, a total number of 12 TFs were strongly enriched (Figures 5(c)-5(d)). CREB3L1 was the top enriched regulator that targeted 17 genes of Module 2. In Module 3, only four TFs were predicted, and SRF was the top enriched regulator that targeted 20 genes of Module 3 (Figures 5(e)-5(f)).

### 3.6. Correlation Analysis of Master Regulators and Modules

**Figure 6:**
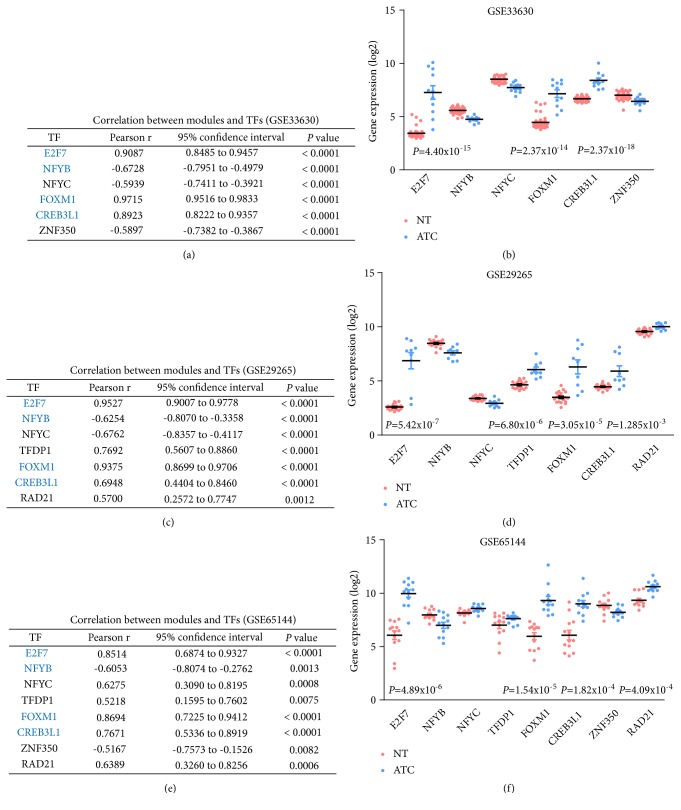
Correlation analysis of master regulators and modules. (a, c, e) Pearson correlation analysis of TFs and modules. Blue fonts indicated consistently correlated TFs in three microarray datasets. (b, d, f) The expression profiles of TFs with Pearson r >0.5 or < -0.5 were retrieved from GSE33630, GSE29265, and GSE65144. TFs, transcription factors.

To elucidate the correlation between master regulators and regulated genes, we compared the expression profile of predicted TFs to expression profile of targeted genes. Basing on the correlation analysis in three microarray datasets, we found that four TFs were coincidentally correlated with key modules in three datasets. Among all candidate genes, E2F7 and FOXM1 showed the highest correlation with the mean expression profile of Module 1 (Pearson r (GSE33630) = 0.9087 and 0.9715, respectively;* P* value < 0.0001) (Figures 6(a), 6(c), and 6(e)). Moreover, E2F7, FOXM1, and CREB3L1 were significantly increased in ATC tissues from three datasets (Figures 6(b), 6(d), and 6(f)). Interestingly, we also found that NFYB, a component of highly conserved transcription factor that bound with high specificity to CCAAT motifs in the promoter regions in a variety of genes, exhibited negative correlation with Module 1 (Pearson r (GSE33630) = -0.6728;* P* value < 0.0001) (Figures 6(a), 6(c), and 6(e)). The expression of NFYB was obviously decreased in ATC tissues among three microarray datasets, which indicated that NFYB may act as a repressor of genes in Module 1 (Figures 6(b), 6(d), and 6(f)). Additionally, CREB3L1 was the only master regulator that significantly correlated with Module 2 (Pearson r (GSE33630) = 0.8923;* P* value < 0.0001) (Figures 6(a), 6(c), and 6(e)). For Module 3, no TFs were identified as master regulators due to the low correlation between TFs and Module 3.

### 3.7. Experimental Validation of Candidate Master Regulators in Thyroid Tissues and Cell Lines

**Figure 7:**
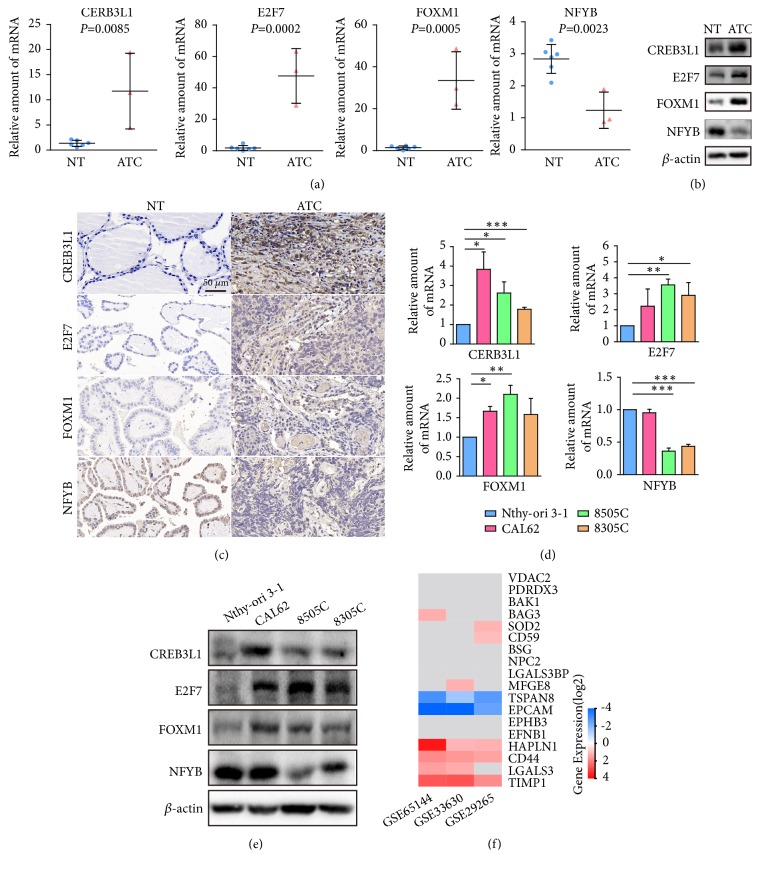
Experimental validation of master regulators in different tissues and cell lines. (a) The mRNA expression levels of CREB3L1, E2F7, FOXM1, and NFYB in normal tissues (n=6) and ATC tissues (n=3). Results were presented as mean ± SD. (b) The expression levels of CREB3L1, E2F7, FOXM1, and NFYB in normal tissue and ATC tissue were detected by western blot. (c) Immunohistochemistry staining of CREB3L1, E2F7, FOXM1, and NFYB in normal tissue and ATC tissue. (d) The mRNA expression levels of CREB3L1, E2F7, FOXM1, and NFYB in Nthy-ori 3-1 and three ATC cell lines. Results were presented as mean ± SD, n=3. ^*∗*^*P*< 0.05, ^*∗∗*^*P*< 0.01, and ^*∗∗∗*^*P*< 0.001. (e) The expression levels of CREB3L1, E2F7, FOXM1, and NFYB in Nthy-ori 3-1 and three ATC cell lines were detected by western blot. (f) Heatmap of the candidate markers in microarray as retrieved from the proteomics data in the literature. Grey block indicated candidates were not significantly changed in microarray dataset. NT, normal thyroid tissues; ATC, anaplastic thyroid carcinoma.

To validate the essential role of CREB3L1, E2F7, FOXM1, and NFYB in ATC progression, we detected their gene expression levels in both human tumor tissues and cell lines. The results showed that CREB3L1, E2F7, and FOXM1 were significantly upregulated in ATC tissues when compared to normal thyroid tissues, while NFYB obviously decreased in ATC tissues (Figures 7(a)-7(b)). Moreover, immunohistochemistry staining indicated that these four TFs were obviously increased or decreased in nuclei of ATC cells (Figure 7(c)). Consistently, we also found that these master regulators significantly changed in three human ATC cell lines as compared with Nthy-ori 3-1 (human thyroid follicular epithelial cell line) (Figures 7(d)-7(e)). Thus, these dysregulated master regulators may be critical for facilitating the aggressiveness of ATC.

To test the consistency of transcriptomics data with the proteomics data in the literature, we examined 18 candidate markers identified by other independent studies [20–23]. The results indicated that only 10 out of 18 genes were significantly changed in microarray datasets. TIMP1, LGALS3, CD44, and HAPLN1 were consistently changed in at least two datasets as compared with the cell line proteomics data by Arcinas et al. [22]. EPCAM and TSPAN8 showed inconsistent trend in ATC tissues, which may result from the heterogeneity between cell line data and tumor tissue data. VDAC2 and BAK1 did not change significantly, which coincided with the results of Mato et al. [21]. According to the results above, more than half candidates matched with current proteomics data. The rest inconsistent candidates may be caused by the heterogeneity of ATC or the complex process from transcription to translation.

## 4. Discussion

Anaplastic thyroid carcinoma (ATC) is an aggressive malignancy with extremely poor prognosis. Its rapid progression and limited therapeutic effect have caused considerable concern. Several studies have attempted to investigate the aberrant gene expression in ATC by high-throughput microarrays. Von Roemeling et al. [9] performed microarray analysis (data deposited as GSE65144) and found that SCD1, a constituent of fatty acid metabolism, was critical for ATC cell survival and proliferation, while they did not conduct GO and KEGG pathway enrichment. The work by Hébrant et al. [7] (data deposited as GSE33630) demonstrated an aggressive molecular switch between PTC and ATC, which supported that ATC may derive from PTC. Recently bioinformatics analysis by Hu et al. [24] also revealed some key pathways in ATC as compared with normal thyroid tissues, while independent cohort validation may be essential for supporting their findings. There are also other literature focuses on the genomic or transcriptomic alteration in ATC leveraging different technologies [25–27]. However, the transcription factor regulatory network in ATC has been little known. In our present study, we used multiple microarrays to identify uniquely changed DEGs between cancerous and normal samples. Integrated bioinformatics analysis and human tissues and cell lines validation were combined to unveil master regulators and key pathways that tightly related to aggressiveness of ATC.

Dedifferentiation is one of the malignant features for ATC. In contrast to the DTC, ATC cells do not remain any of the biological features or functions of normal follicular cells, such as thyroglobulin synthesis, TSH dependence, and iodine uptake [28]. Among the top 20 downregulated DEGs, near half DEGs were involved in thyroid differentiation and function maintaining. DEGs like thyroglobulin (TG), thyroid peroxidase (TPO), thyrotropin receptor (TSHR), dual oxidase 2 (DUOX2), iodothyronine deiodinase 1 (DIO1), solute carrier family 26 member 4 (SLC26A4, also known as pendrin), and iodotyrosine dehalogenase 1 (IYD-1) are critical for biosynthesis, storage, and secretion of the thyroid hormones T3 and T4 [29]. Other DEGs including FOXE1 and NKX2-1, PAX8, and HHEX (significantly changed but not in the top 20 downregulated DEGs) are thyroid transcription factors that are fundamental for thyroid differentiation and for maintaining the functional differentiated state of the adult thyroid [29]. Though loss of differentiation in ATC has been generally recognized, our current study provided a better insight into the transcriptomic landscape of thyroid cancer dedifferentiation.

ATC is extremely malignant with significant changes in both intracellular signal pathways and tumor microenvironment. Pathway enrichment showed that ECM-receptor interaction, cell cycle, PI3K-Akt signaling pathway, protein digestion and absorption, phagosome, osteoclast differentiation, focal adhesion, p53 signaling pathway, staphylococcus aureus infection, and leishmaniasis were potentially activated in ATC. Aberrant activation of PI3K-Akt signaling pathway and p53 signaling pathway was tumorigenic for thyroid gland [30]. Both pathway mutations were higher prevalence in ATC [4, 31]. Alterations in these signals were correlated with tumor recurrence, lower survival rates, and poor prognosis in thyroid cancer patients [32]. Gene mutation and aneuploidy in tumor cells also lead to the generation and aggregation of misfolding protein, which could induce cell apoptosis. Activation of the protein digestion and absorption pathway or phagosome could clean misfolding protein and prevent tumor cells from apoptosis [33]. Additionally, ECM-receptor interaction and focal adhesion are also essential for tumor invasion and migration [34]. The interactions between extracellular matrix and tumor cells lead to a direct or indirect control of cellular activities such as cell motility, adhesion, migration, differentiation, proliferation, and survival. Strikingly, we found that the collagen family members were widely involved in ATC progression. Ten collagen genes, including COL1A1, COL1A2, COL3A1, COL5A1, COL5A2, COL5A3, COL6A1, COL6A2, COL6A3, and COL11A1, were commonly enriched in ECM-receptor interaction, PI3K-Akt signaling pathway, protein digestion and absorption, and focal adhesion. Type I collagen was upregulated in PTC and expressed at the highest levels in PDTC and ATC. Fibroblast-derived type I collagen could enhance cancer cell motility and facilitate the progression of thyroid cancers [35]. Even though type I collagen has been fully studied in ATC, the role of types III, IV, VI, and XI collagens in ATC progression still remains mystic. Further studies were needed to unveil their functions and to explore their values as therapeutic targets.

By constructing the PPI network and regulatory network, three key modules and four master regulators were identified. Module analysis showed that 8 out of 10 hub genes were involved in Module 1 network, including TOP2A, CDK1, CCNB1, BIRC5, CCNA2, MAD2L1, CDC20, and BUB1. Function annotation indicated that these genes were essential for cell cycle, cell division, and DNA replication. Uncontrolled cell proliferation and DNA replication have been considered as one of the hallmarks of cancer [36]. Our regulatory network analysis indicated that E2F7, FOXM1, and NFYB were master regulators of Module 1, and most hub genes above were targeted. E2F7 is an E2F transcription factor that is involved in various processes such as angiogenesis and DNA damage response. Studies showed that E2F7 could promote cancer cell proliferation, migration, and metastasis [37, 38]. Liu et al. found that the feedback loop between miR-26a and E2F7 could also promote tamoxifen resistance in ER-positive breast cancer [39]. However, the role of E2F7 had not been specifically implicated in ATC. FOXM1 is a member of the forkhead box family that participated in cell proliferation, chromosomal stability, angiogenesis, and invasion, while its role in ATC has not been fully determined. We found FOXM1 significantly increased in ATC and highly correlated with Module 1. Accordingly, results from the study by Bellelli et al. [40] also identified FOXM1 as a molecular determinant of the mitogenic and invasive phenotype of anaplastic thyroid carcinoma [40]. Further study was needed to elucidate the pivotal role of FOXM1 during ATC progression. Notably, we also found that the NFYB (nuclear transcription factor Y subunit beta) was a potential repressor of Module 1 and was downregulated in ATC. NFYB was component of the heterotrimeric transcription factor (NF-Y) and NF-Y can function as both an activator and a repressor depending on its interacting cofactors. The tumor suppressor p53 negatively regulated CHEK2 gene transcription through modulation of NF-Y function, and this regulation was important for reentry of cells into the cell cycle after DNA damage is repaired [41]. However, the regulatory function of NFYB in ATC progression still remained mystic.

CREB3L1 was the master regulator that specifically targeted genes in Module 2. More than 70% genes of Module 2 consisted of collagen family members, including ten collagen genes that shared by four key pathways above. CREB3L1 is activated by endoplasmic reticulum (ER) stress. Knockdown of CREB3L1 in glioma cells resulted in decreased expression of extracellular matrix proteins and attenuated ER stress response [42]. Moreover, recent study indicated that CREB3L1 was a key downstream mediator of PERK-driven metastasis in breast cancer [43]. Even though limited studies determined the role of CREB3L1 in ATC progression, transcriptional regulation of collagen family by CREB3L1 holds promise for it to be the candidate target of ATC.

## 5. Conclusions

In conclusion, our current study aimed at identifying master regulators involved in the progression of ATC via integrated bioinformatics analysis. This study provided several novel master genes and pathways for future investigation of the mechanisms underlying ATC. This comprehensive bioinformatics analysis sheds light on the pathogenic mechanism investigation and target mining, while their preclinical and clinical values require further validation. Moreover, the transcriptional regulation of candidate genes by TFs and the activation manner of TFs need further experimental validation in ATC.

## Data Availability

The microarray datasets GSE33630, GSE29265, and GSE65144 can be retrieved from Gene Expression Omnibus (GEO, http://www.ncbi.nlm.nih.gov/geo/) database in the National Center for Biotechnology Information (NCBI).

## Supplementary Materials

Supplementary Materials The tables with the full list of detected genes in each dataset along with their intensities, the fold changes, and the* P* values were uploaded in Supplementary Materials. Moreover, the Relative Log Expression (RLE) plots of GSE33630, GSE29265, and GSE65144 were also conducted and uploaded (See Figures S1–S3 in the Supplementary Materials for comprehensive image analysis).
