# Supplementary figures and images for: Integrated Bioinformatics Analysis of Master Regulators in Anaplastic Thyroid Carcinoma
Source: Biomed Res Int. 2019 Apr 28;2019:9734576. doi: 10.1155/2019/9734576 (PMC6512074; doi:10.1155/2019/9734576)

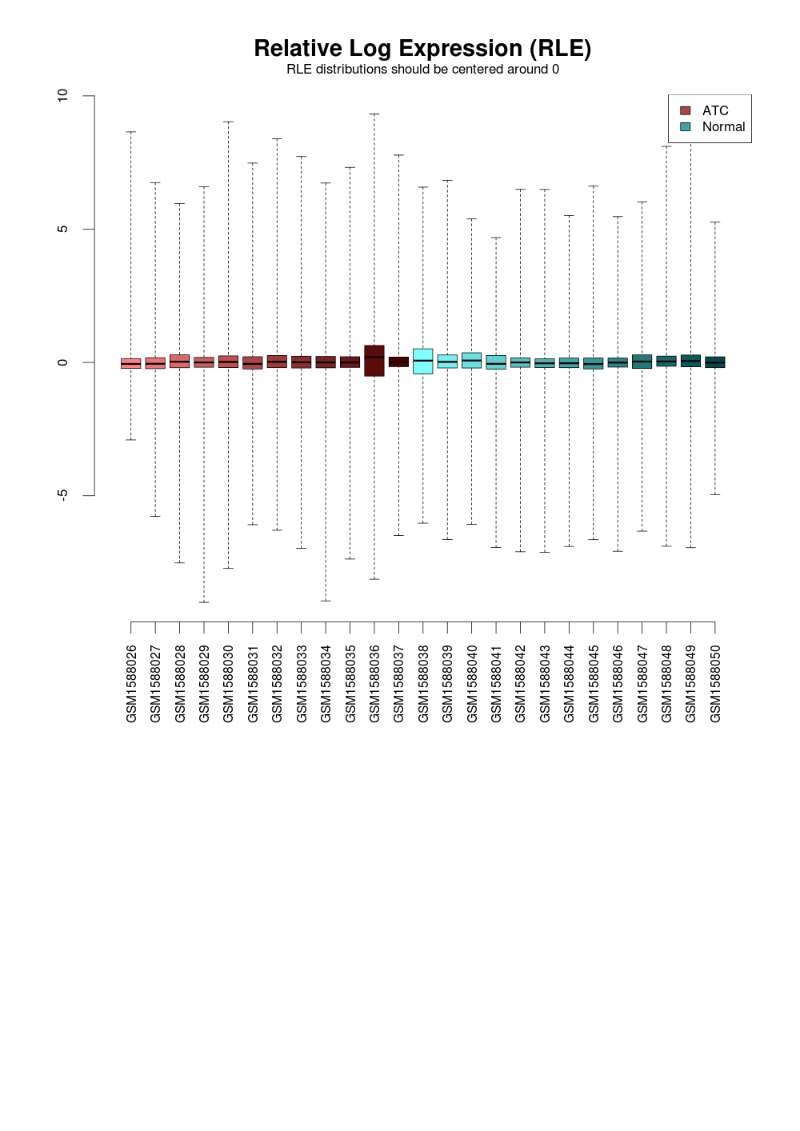

Supplement: Supplementary Materials — The tables with the full list of detected genes in each dataset along with their intensities, the fold changes, and the P values were uploaded in Supplementary Materials. Moreover, the Relative Log Expression (RLE) plots of GSE33630, GSE29265, and GSE65144 were also conducted and uploaded (See Figures S1–S3 in the Supplementary Materials for comprehensive image analysis). [file 9734576.f1.zip › Figure S1 RLE plot of GSE65144.jpg]

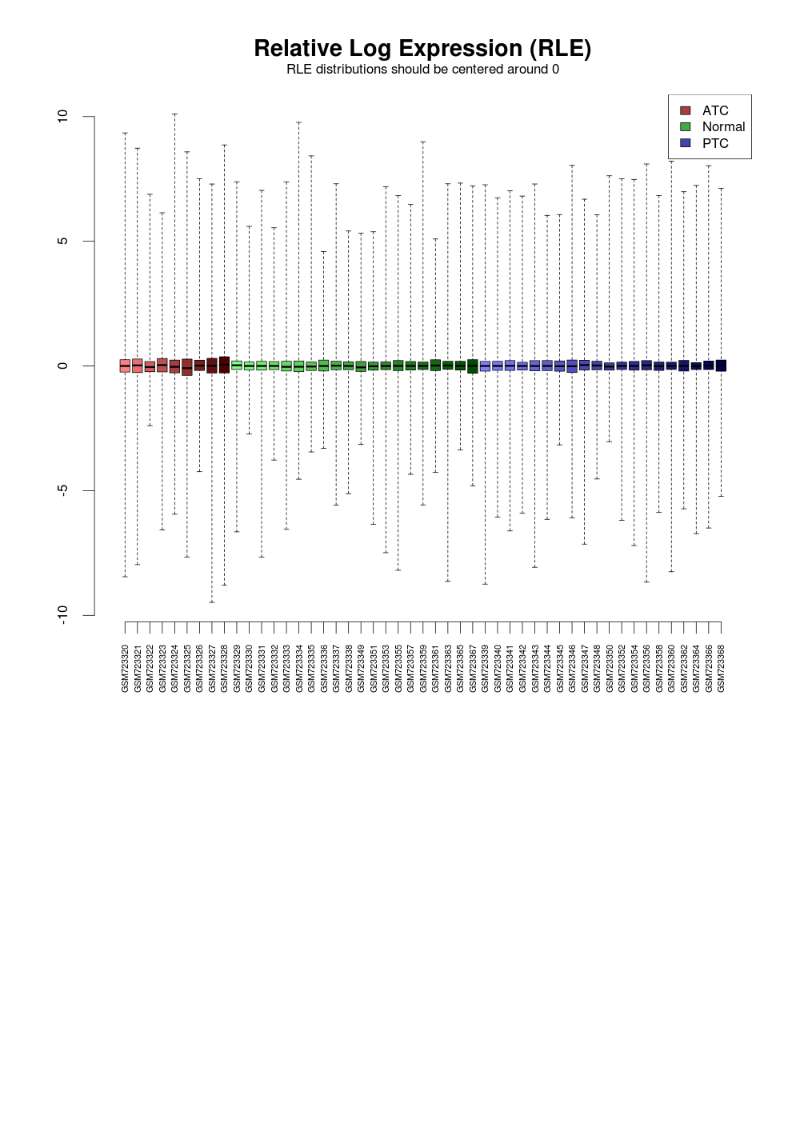

Supplement: Supplementary Materials — The tables with the full list of detected genes in each dataset along with their intensities, the fold changes, and the P values were uploaded in Supplementary Materials. Moreover, the Relative Log Expression (RLE) plots of GSE33630, GSE29265, and GSE65144 were also conducted and uploaded (See Figures S1–S3 in the Supplementary Materials for comprehensive image analysis). [file 9734576.f1.zip › Figure S2 RLE plot of GSE29265.jpg]

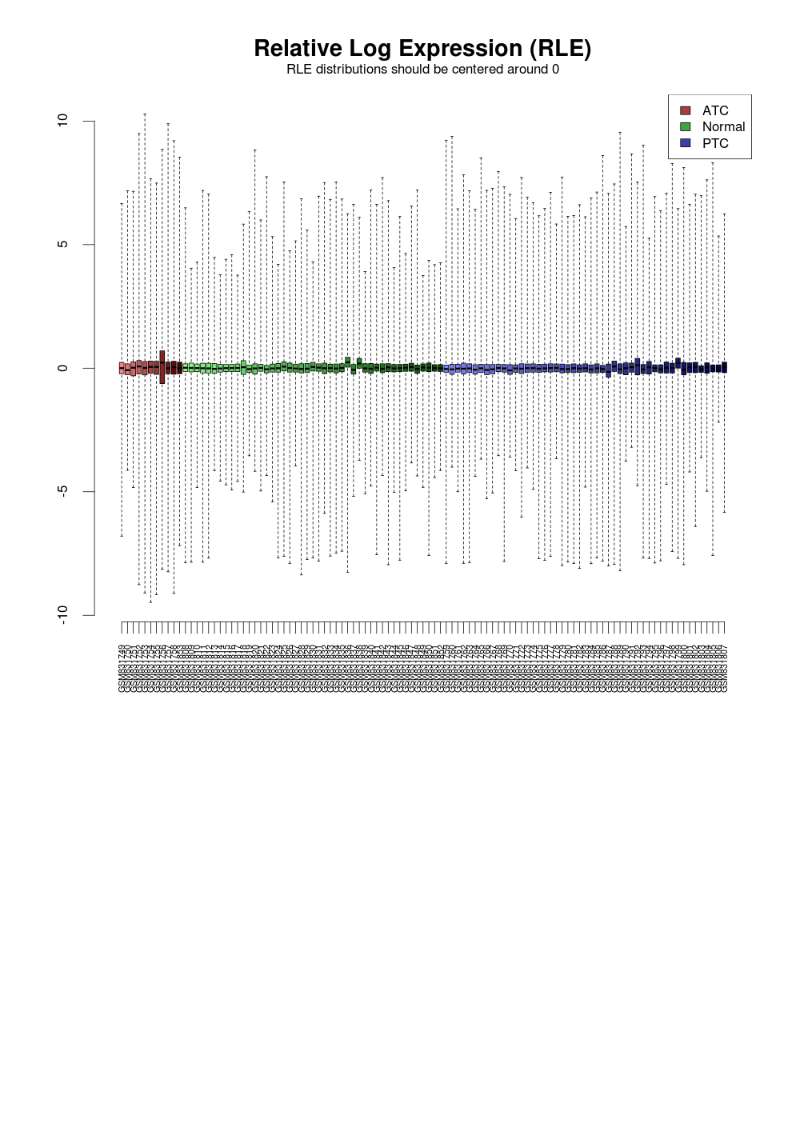

Supplement: Supplementary Materials — The tables with the full list of detected genes in each dataset along with their intensities, the fold changes, and the P values were uploaded in Supplementary Materials. Moreover, the Relative Log Expression (RLE) plots of GSE33630, GSE29265, and GSE65144 were also conducted and uploaded (See Figures S1–S3 in the Supplementary Materials for comprehensive image analysis). [file 9734576.f1.zip › Figure S3 RLE plot of GSE33630.jpg]
